# The Human Mind, Considered in Some of Its Medical Aspects


**Published:** 1850-04-01

**Authors:** J. Bower Harrison


### THE HUMAN MIND CONSIDERED IN SOME OF ITS MEDICAL ASPECTS.*

BY J. BOWER HARRISON, M.R.C.S.L.

There is nothing which is more common than to hear people speak of possessing a knowledge of human nature. It is usual to signify by this a mere acquaintance with the vices of mankind, which does, indeed, form one part of this knowledge, but it is not all. What is called a knowledge of human nature is said to be acquired only by mixing with the world, which implies that a real intimacy with the workings, tendencies, and capabilities of the human mind is industriously kept back in our literature, and that by the general consent of mankind it is deemed better that a great part of the world should be ignorant of the true position of their fellow beings. Many of those who are teachers of religion and morality, have certainly derived their ideas on this subject from sources which show, in point of fact, a complete ignorance of human nature, or, at least, the studious *disguisement* of such knowledge.

The reserve, which is common and proper in society, may screen from the unreflecting, and those little conversant with life, much of the real nature of the human mind.

The prescribed forms of living, of speaking and writing, and the conventional usages of mankind, have given an artificial hue to our actions and language, and thus the words of the great dramatist have become literally true, that "all the world is a stage, and all the men and women merely players." The true character of the human mind in its more intimate workings, is little confessed in our literature, and little acknowledged in our laws. Unfortunately, the real appreciation of the human mind (with regard to its actual state, and not its abstract consideration) has scarcely appeared to fall within the province either of the moralist or metaphysician, and has remained a kind of neutral ground, uncultivated because unclaimed. It does not seem the part either of the metaphysician or the psychologist to follow out all the fine workings of the human mind, and disclose its hidden secrets. That knowledge which is sometimes designated a *knowledge of the world*, approaches, perhaps, nearest to the subject in question, but is seldom possessed by those who are either desirous or capable of systematizing their information, and thus, much of what is really known of our common nature is lost both to the philosopher and philanthropist. In the writings of some of our novelists, it is true, we find a considerable amount of this species of information, but it is for the most part rendered sub-

* Read at a *Conversazione* of the Royal Manchester Institution.

servient only to the entertainment of the reader, and not in every case calculated for his edification. The writings of Fielding, Smollett, Cervantes, and Le Sage, with many others of more modern date, amongst which I may mention Mr. Charles Dickens, abound in judicious reflections on men and manners, and discover no little penetration into character and mind. After all, however, there is an evident deficiency in published or recognised information of unsophisticated humanity; and hence the surprise or affected surprise with which it is common to view what are called the inconsistencies of human nature. That certain infirmities of mind are not incompatible with genius it may be common to admit, but when we approach the subject closer, we shall find the poetic temperament more frequently than not clouded with insanity, or touched with perversity. The same bright spirit which rejoices in the sunshine of nature is not less sensitive of the gloom. There is often a foreboding of evil, and often the imagination will heighten the calamities which are really present, until a thousand heartrending associations grow up in the fertility of the mind.

The consideration of the human mind, then, in what may be called its medical aspects, seems to me one which has not yet sufficiently engaged the general attention of mankind, and I wish on the present occasion to bring it before your notice, as a subject which is neither devoid of interest nor advantage.

The effect of temperament may first claim our attention. I shall not, however, confine myself to the divisions which strictly medical writers have formed on this subject, reserving to myself the freedom of the popular acceptation of the word.

Physical Constitution.—The constitution of the body as to its bulk and general aspect, as well seen in the countenance as in the conformation of other parts, is known to be connected with accompanying peculiarities of mind which are of every-day observation. There is an asperity in the countenance and figure of some people which immediately strikes us, and we feel a sort of intuitive dislike. There are others whose open countenance and portly bearing tell at once of generous feelings and easy dispositions. Every one remembers the passage in the play of Julius Cæsar :—

> "Would he were fatter; but I fear him not,—
> Yet if my name were *liable* to fear,
> I do not know the man I should avoid
> So soon as that *spare* Cassius."

The Sanguine Temperament, as the very name implies, is characterised by a constitution in which the blood-vessels are predominant; the face is red, and easily flushed; the complexion often light; the temper hasty; the actions quick. Those who have this temperament in a marked form often act by impulse ; they are restless, and have difficulty in waiting for the result of their undertakings. The mind is often too easily excited to allow of a proper operation of its workings ; the ideas flow too quickly, and in a little while become confused. Such persons, when called upon in exciting circumstances, become embarrassed, are what they call nervous, and lose self-possession or act to disadvantage.

The character of Peter in the sacred writings gives us a good example of this sanguine temper; we find him saying to his Lord, "Though all men shall be offended because of thee, yet I will never be offended; I will lay down my life for thy sake:" and again, "though I die with thee, yet will I not deny thee." But as in many other sanguine people the feeling wanted the support of a steady equanimity. When Jesus was betrayed, and Peter found himself accused of being in his company, he denied any knowledge of him, and when he found that he was known by his accent, ("surely thou also art one of them, for thy speech betrayeth thee"), "he began to curse and to swear, saying, I know not the man." Such is often the fate of the warm protestations of sanguine people. The emotion which attends the expression of their sentiments gives the complexion to them whilst it exists, but it is not enduring, and is succeeded by other emotions, which for the time often overcome the better feelings of the heart. This sanguine temperament gives what we call the generous character; the warmth of temper which, in moderation, we rather admire as heightening the affections, and giving joy and gladness: but it has also its sorrows and its errors. There is the quick resentment and the passionate grief, and these are the dark aspects, which are full of tears and lamentation. Hamlet, you know, says to Horatio :—

"Give me the man that is not passion's slave,
And I will wear him in my heart's core;
Aye, in my heart of hearts as I do thee."

In sanguine people, the train of thoughts appears to be much quicker than in others. This is attended with certain advantages, but corresponding disadvantages. Such minds are imaginative—what is called ingenious. The associations are not too strong to forbid erratic thoughts; relations are often readily perceived, but there is less exactness. The memory is, perhaps, less retentive, and the speed of the thoughts leads often to confusion. They crowd on one another until indistinctness arises. This indistinctness is sometimes painful.

This kind of mind is what is called passionate; hence the term *hasty*, which may be well used as indicative of a too rapid procession of thoughts. The mind cannot well operate in this hurried state. The ideas flash across, but they light up too fiercely a train of associations to admit the suggestions of other minds.

The brain is, perhaps, over sensitive, and its powers too readily exhausted, and too intense whilst in operation.

The Lymphatic Temperament is quite the reverse of the sanguine. Those who have this temperament are not easily moved, or excited; the skin is pale. Such persons are better calculated to undergo anxieties, or meet the exigencies of sudden emergencies. The mind has not those quick sympathies, or brilliant imaginings, which distinguish a warmer temperament.

There is a steady, cool calculation, which is more adapted to worldly prosperity; feelings less readily wounded, and less apt to betray themselves in the excitement of life. Some possess this in a surprising degree, and many assume it. Many cover a natural sensibility by the forms and usages which polite life has invented, and studiously avoid showing the natural burst of affection, or the bitter outpourings of grief.

It is well, in some degree, that this should be done; but let not nature be altogether destroyed, and all the fine sensibilities crushed, under an artful and sophisticated mannerism.

I think Mr. Dawson tells a story of a cockney, who visited the beautiful and romantic scenery of the Alps, and being asked his impression of a sublime prospect, which suddenly burst on his view, said, that he must confess "it was a well got up thing." This is the school of fashion, which gives us two fingers to touch as a welcome after the absence of as many years.

In some, the operations of the mind are so slow that they seem to stagnate, and pass into reverie. The extreme of this condition lapses into a state of imbecility and fatuity; but a certain degree of apathy has its advantages. The mind has time to act; there is no over-crowding of ideas—no hurry of thought. There is more continuance of application—less impatience of consideration. The *affections* do not trouble the operations of the mind. There are not those quick sympathies which have relations with everything that passes.

There are some people, again, in whose constitution the nervous system seems to have an unnatural preponderance; the mind seems to be constantly in a restless state, and to wear out the body in a ceaseless activity, or in the struggles of overweening ambition. Such characters are ever pressing on in the competition of social life; often a prey to cares and anxieties, which follow each other in a never-ending succession. "*After life's fitful fever*," they indeed "*sleep well.*"

Hypochondriacal feelings are, no doubt, in a great measure connected with constitution or temperament. Melancholy is much more common than is generally conceived, and may be, as I have already hinted, perhaps in some degree inseparable from a mind which highly appreciates the beautiful, has quick sympathies with all around, and a thoughtful regard to the possibilities, and even probabilities of a changing world. On this account it has always been considered by the poets as a poetic feeling, and though this view may have attracted the sneers of some, or the ridicule of others, there is a certain truth in the opinion. Ben Jonson alludes to this notion, with respect to melancholy, as an accompaniment of sensibility, in his "*Every Man in his Humour.*"

"*Stephen.* Ay, truly, sir, I am mightily given to melancholy.

"*Matthew.* Oh! it's your only fine humour, sir; your true melancholy breeds your perfect fine wit, sir. I am melancholy myself divers times, sir; and then do I no more but take pen and paper presently, and overflow your half a score or a dozen of sonnets at a sitting."—*Act III., Scene* 1.

Again :—

"*Ed. Knowall.* . . . . What think you of this, coz?

"*Stephen.* Why, I do think of it; and I will be more proud and melancholy and gentlemanlike than I have been, I'll insure you."—*Act I., Scene 2.*

In Scott's "Diary" (May, 1827,) he says :—"Imagination renders us the victim of occasional low spirits. All belonging to this gifted, as it is called, but often unhappy class, must have felt, but for the dictates of religion, or the natural recoil of the mind from the idea of dissolution, there have been times when they would have been willing to throw away life, as a child does a broken toy. I am sure I know one who has often felt so."

A disposition to melancholy is by no means, as might at first be imagined, necessarily indicated by a grave and staid deportment.

In a large proportion of instances, it is even coupled with an exuberance of spirits, which would seem to promise a perpetual sunshine of cheerfulness.

The mind, however, which is alive to joy, is also, and perhaps equally, alive to sorrow; and often passes by quick transitions from the one to the other.

Our great dramatist, in mingling the ludicrous with the tragic, has evidently shown his acquaintance with this tendency of the human mind. Byron has happily blended in some of his most successful passages the sublime and the ridiculous, thereby greatly heightening the effect of both. In his "*Corsair*," however, he has directly touched upon this peculiarity of the mind, where he says—

"Strange though it seem, yet with extremest grief
Is linked a mirth,—it doth not bring relief.
That playfulness of sorrow ne'er beguiles,
And smiles in bitterness,—but still it smiles."

In Dr. Currie's Life of Burns, allusion is made to the combination of melancholy and gaiety in the character of that extraordinary poet.

Notwithstanding the gaiety of Burns' writings, he was constitutionally a melancholy man, and was subject "to those depressions of mind, which are perhaps not wholly separable from the sensibility of genius, but which, in him, arose to an uncommon degree." . . . . "Such a disposition is far from being at variance with social enjoyments. Those who have studied the affinities of mind, know that a melancholy of this description after a while seeks relief in the endearments of society, and that it has no distant connexion with the flow of cheerfulness, or even the extravagance of mirth."*

In a letter from Sir Walter Scott to his daughter in-law, the following mention is made of Mathews, the comedian. "I am glad you like my old acquaintance, Mathews. Some day I will make him show his talent for your amusement in private—for I know him well. It is very odd—he is often subject to fits of deep melancholy."†

From these cursory remarks on melancholy, I may proceed to speak of some particular states of mind, which attend the same pecu-

* P. 105. † Vol. iv. p. 10, Lockhart's Life.

liarity of temperament, and though not incompatible with mental sanity, are yet to be considered as bordering upon derangement.

Some of the illustrations I shall bring forward are from works of poetry, but may be considered, nevertheless, as expressive of actual facts, because I have scrupulously avoided making use of them where the subject had need of graver support; so that in effect they are rather introduced to relieve and exemplify the matter, than to substantiate it. The curious feelings to which I allude, exist in various degrees of intensity in different people. They cannot be considered as common to everybody, and are far more frequent or powerful in the imaginative and eccentric, than in others. Yet I should conceive that they prevail to a greater extent than is commonly supposed.

Amongst this class of feelings may be placed the disposition which is sometimes manifested by those who stand on precipitous cliffs to cast themselves down. This seems suggested by a kind of insane impulse, which is dreaded by the very person who indulges it.

Scott beautifully alludes to such a feeling in "The Lady of the Lake."

> "There are, who have at midnight hour,
> In slumber scaled a dizzy tower,
> And on the verge that beetled o'er
> The ocean tides incessant roar,
> Dreamed calmly out their dangerous dream,
> Till wakened by the morning beam;
> When dazzled by the eastern glow,
> Such startler cast his glance below,
> And saw unmeasured depths around,
> And heard unintermitted sound,
> And thought the battled fence so frail,
> It waved like cob-web in the gale;
> Amid his senses giddy wheel
> Did he not desperate impulse feel
> Headlong to plunge himself below,
> And meet the worst his fears foreshow?"—*Canto II.*, 22.

The same is alluded to by Shakspere in his "Hamlet," when he is cautioned by Horatio not to follow the Ghost,—

> "What, if it tempt you towards the flood, my lord,
> Or to the dreadful summit of the cliff
> That beetles o'er his base into the sea;
> And there assume some other horrible form,
> Which might deprive your sovereignty of reason,
> And *draw you into madness?* Think of it,—
> The *very place* puts toys of desperation,
> *Without more motive*, into every brain,
> That looks so many fathoms to the sea,
> And hears it roar beneath."

This, though fiction, is of course true to nature. We know how many have jumped from monuments, and given way to an impulse to suicide created by circumstances. Many instances of the same kind might be brought together, not merely from our classical writers, but from the pages of our journals and newspapers. The cases of suicide which seem to have been suggested by the contemplation of frightful precipices—deadly poisons, or dangerous weapons, may be considered under this head. Hence the importance of avoiding evil thoughts and tampering with dangerous ideas. The same kind of feeling sometimes urges people to contemplate scenes of suffering and distress, which are painful to be witnessed, or pushes them on to acts which they at the same time dread. This tendency is also well alluded to by the great poet of nature in the scene in "Romeo and Juliet," where the latter contemplates her anticipated position on the tomb of all the Capulets:—

> "How if, when I'm laid into the tomb,
> I wake before the time that Romeo
> Comes to redeem me?
> * * * *
> Alack! alack! shall I not be distraught,
> Environed with all these hideous fears?
> And madly play with my forefathers' joints,
> And pluck the mangled Tybalt from his shroud?
> And in this rage with some great kinsman's bone,
> As with a club, dash out my desperate brains?"

This insane feeling is here well portrayed, where the very speaker has been previously expressing her horror of that dreadful place, where—

> "Bloody Tybalt, yet but green in earth,
> Lies festering in his shroud."

There are feelings sometimes which are contradictory in their nature and objects. The mind is strangely perplexed in a war of emotions—emotions which are cherished whilst they are painful. Actions are not always dictated by the simple feelings which seem to have given them birth. How many persecuting spirits have wept for those they have persecuted. There are those whose feelings of mercy and forgiveness have struggled hard and yet yielded to pride and an ignoble revenge. Such contradictory feelings do indeed partake of an insane character, and there is a fine significance in the words of the poet,—

> "That to be wroth with those we love,
> Doth work like madness on the brain."

I bring forward these remarks on such states of mind, because I think it of great importance (as they really do exist) that they should be at once deprecated as unhealthy and insane feelings; and in order if possible to make it appear that the proper control of the mind, which constitutes virtue, is in reality the most conducive to a sound understanding. Let it not be considered, however, that I mean to assert that mental derangement is necessarily the result of vice—it is more often the cause. To resume, however. In the autobiography of the celebrated Goëthe, he makes reference to this most unfortunate contention of feeling.

Goëthe says,—

"I accordingly fell into that evil disposition of mind which often misleads us so far as to make us find a pleasure in tormenting those whom we love; and I abused the fondness of a young female by tyrannical and arbitrary caprices. Secure of the affection of Annette, and of her anxiety to please me, I vented on her all the ill-humour that the failure of my poetical essays, the apparent impossibility of doing myself honour by them, and everything else that occurred to vex me. I poisoned our best days by groundless and unworthy jealousies. She long endured all these follies with angelic patience; but I had the cruelty to tire it out."*

Hazlitt observes, that men often "take a perverse delight in acting, not only contrary to reason, but in opposition to their own inclinations."†

Again and again I may say that the contemplation of these curious workings of the mind should lead us to see the importance of attending to its due regulation, and lead us to regard improper feelings as insane impulses which are to be dreaded as disease.

The peculiar notions which some eminent men entertain, probably against their better judgments, may be also just alluded to. Scott speaks of a feeling of *pre-existence* in his Diary (Feb. 17):—

"A hard day of working, being I think eight pages before dinner. I cannot, I am sure, tell if it is worth marking down, that yesterday, at dinner time, I was strongly haunted by what I would call the sense of pre-existence—viz., a confused idea that nothing that passed was said for the first time; that the same topics had been discussed, and that the same persons had stated the same opinions on them. . . . . . There was a vile sense of want of reality in all I said and did. . . . . . Something of this insane feeling remains to-day, but a trifle only."‡

This feeling of pre-existence is a sort of poetical feeling; I mean most frequently met with in persons of highly imaginative moods, or engaged in metaphysical pursuits. Dickens mentions in his Pictures from Italy his first sight of Ferrara in the following terms:—

"On the foreground was a group of silent peasant girls leaning over the parapet of a little bridge, looking now up at the sky, now down into the water; in the distance a deep bell; the shadow of approaching night on everything. If I had been murdered there on some former life I could not have seemed to remember the place more thoroughly, or with more emphatic chilling of the blood; and the real remembrance of it acquired in that minute is so strengthened by the imaginary recollection, that I hardly think I could forget it."§

Shelley states "that whilst walking with a friend in the neighbourhood of Oxford, a scene presented itself which he remembered long ago to have seen *in a dream*."|| Mrs. Shelley remarks on this, "I remember well his coming to me pale and agitated to seek refuge in conversation from the fearful emotions it excited."

Many of us may have a sort of feeling of reminiscence of things which we in reality see for the first time, probably because they

* P. 218, vol. i. † Characteristics, p. 82. ‡ P. 114, vol. vii.
§ P. 102. || Shelley's Memoirs, p. 135.

awaken a train of thought similar to what other objects may have previously excited.

From these desultory remarks on the characters of mind which accompany the more highly, and may be unduly, developed powers of the imagination, I may proceed to speak of the connexion of bodily disease with mental peculiarity.

Disease.—Every one knows the effect of disease on the human mind in rendering it a prey to uneasy feelings and capricious changes. Happy, indeed, is he who has no reminiscences of weary hours spent in midnight vigils, or long days of unrest, in which the mind refused to take its wonted pleasure in occupation or literature. We are, perhaps, too apt to regard our minds as above the influence of circumstances, and altogether dependent on self-control and voluntary effort. Disease may teach us that the mind itself is the gift of a superior power, and its very complexion dependent on causes which we cannot command.

Shakspere, as you all well know, makes a happy allusion to the effect of disease in enervating the mind, in Cassius's speech in contempt of Cæsar :—

> " He had a fever when he was in Spain,
> And when the fit was on him, I did mark
> How he did shake. 'Tis true, this god did shake :
> His coward lips did from their colour fly;
> And that same eye, whose bend doth awe the world,
> Did lose his lustre ! I did hear him groan ;
> Ay ! and that tongue of his, that bade the Romans
> Mark him, and write his speeches in their books,
> Alas ! it cried, Give me some drink, Titinius,
> *As a sick girl.*"

Apoplexy and palsy bring the finest minds to imbecility The sufferer yields to fits of passion, and readily " dissolves in tears," in alternate succession, and thus remains an object for pity and the tolerance of friendship, until another attack completes the ruin, and leaves him a miserable wreck—the shadow of his former self. " By this distemper," Sir Henry Halford tells us, " the talents of the great Marlborough were confounded." The most serene and powerful minds are troubled by disorders of this nature,—curious aversions, fits of irritation, and whining imbecility, take the place of easy and kindly dispositions ; so that our very virtues are dependent on the sufferance of a superior power.

The languor and lassitude which accompany biliary derangements are well known and of common remark. The peculiar perverse states of mind which attend various forms of hysteria, furnish us with some of the most curious instances of human weakness which can possibly engage the attention. The effects of indigestion in creating frightful dreams, have been experienced by us all. How rejoiced we are, after suffering from the most terrible nocturnal alarm, to awake, and " find it all a dream." Perchance a bull pursues us, and our limbs refuse to move,—a wild and disgusting animal fastens its teeth on us, and escape is in vain,—an immense load bears on us, and instant suffocation is impending. Sterne, in his Sentimental Journey, illustrates the effects of disease on the mind in his usual striking manner :—

"The learned Smelfungus travelled from Boulogne to Paris, from Paris to Rome, and so on. But he set out with the spleen and jaundice, and every object he passed by was discoloured or distorted. He wrote an account of them, but 'twas nothing but the account of his miserable feelings. I met Smelfungus in the grand portico of the Pantheon,—'Tis nothing but a huge cock-pit, said he. * * * I popped upon Smelfungus again at Turin in his return home, and a sad tale of sorrowful adventures had he to tell. * * * He had been flayed alive, and bedeviled, and used worse than St. Bartholomew, at every stage he had come to. 'I'll tell it,' cried Smelfungus, 'to the world.' 'You had better tell it,' said I, 'to your physician.'"

It is well known that age induces great mental changes. We are all willing to suppose that we grow wiser as we grow older, and it is to be hoped that this is really the case; but so far as our morality is concerned, we should bear in mind that we forsake many of our vices only because the temptation no longer exists. We "*put away childish things*" when we cease to have the desires of childhood ; and the vices of youth would often come back in advanced life if we could bring back the fresh feelings of youth, and all the impulses which first took possession of our souls. How much such considerations should make us humble, charitable, and forgiving; should render us distrustful of our own virtue, and compassionate for the feelings of others, and make us thankful to *Him* from whom all goodness proceeds.

Delicate states of health often induce, or are connected with, states of mind which seem too refined for the common intercourse of life. There is often a precocity of mind in delicate children which far surpasses in nicety of feeling the ideas of those more advanced in age. They who have reminiscences of their early sick beds will know how little their own ideal creations were understood, and how little known were the deep recesses of their infant minds. The practical in after life takes the place of the poetical, and the fine sensibility shrinks and withers in the rude competition of actual life, and in reality "concealment, like a worm i'th' bud, feeds on the damask cheek."

Debility of body is often, in fact, generally, productive of great mental susceptibility, and sometimes irritability of mind. Shakspere says—"Conceit in weakest bodies strongest works." People who have robust health, and have always enjoyed such, can enter little into the sentiments of others, nor appreciate the feelings which accompany feeble and imperfect states of health, or constitutions of greater delicacy and refinement. Shelley says, "I know not the internal constitution of other men, I see that in some external attributes they resemble me; but when, misled by that appearance, I have thought to appeal to something in common, and unburden my inmost soul, I have found my language misunderstood, like one in a desert and savage land."*

INSANITY.—In considering the human mind, in its medical aspects, it might naturally enough be supposed, that the subject of insanity would form a principal part. I shall not, however, for many reasons enter upon it in this place, further than to touch upon those lighter, or more partial derangements, which are less commonly made matter of observation or remark.

It has often appeared to me, that much good might arise from the consideration of mental sanity, on a finer scale than has hitherto been afforded. The condition of the mind may not be properly healthy, without being betrayed into those complete aberrations which shock the observer, or call for the interference of friendly restraint. The subject has its relations, both to morals and jurisprudence; but there is, perhaps, no education which so fits the mind to enter on its consideration, as that which is prescribed by the study of medicine.

When we contemplate our species in the workings of great and good minds, we cannot fail to feel proud of a humanity which is capable of achieving vast designs, and compassing mighty undertakings. The names of Newton and Locke inspire us with a kind of reverence for the capabilities of our nature. We then, indeed, perceive, that there is "a divinity which stirs within us, and indicates eternity to man." How high and godlike is the mind which can scan its own operations, sit in judgment on itself, review the events of past ages, and anticipate the future progress of societies and nations. It would be a pleasing task to dwell on the greatness of the human mind. We see it in the honest and sturdy Franklin—the humble and philosophic Dalton—the persevering and indefatigable Priestly—the bold and imaginative Davy—the philanthropic Howard—the laborious and highly-gifted Hunter. If we turn from this picture of the more lofty style of man, and look to those aberrations of the intellect which we find in the receptacles for the insane, we must feel a humiliation at least equal to our previous gratification. Here we have the drivelling idiot—the raving madman—the moping melancholic—the incoherent dreamer—and the timid and suspicious hypochondriac. Nor is it a correct opinion, that the division between the intellectual and insane portion of mankind is so broad as it appears on a cursory consideration. That there are minor aberrations of mind which do not come within the limits of actual madness, there can be no doubt; nor is real insanity so far dissevered from the more healthy operations of the mind, as to be altogether incompatible with intermissions of extraordinary brightness.

It is well known that many of our eminent writers, especially those of an imaginative kind, were hypochondriacs. The unfortunate Cowper and Collins were of this class; and Chatterton and Haydon, it will be remembered, destroyed themselves under its influence. The

Rev. Robert Hall had periods of actual insanity, and the elegant Charles Lamb probably escaped from his sister's fate, only by the necessities of his own position. Whitbread, Romilly, Londonderry, and Calcraft, put an end to their own existences; but the list is too great to particularize, and it is enough to know, that learning and genius are not removed from the worst of human calamities.

The fate of the unfortunate Swift is too well known to need comment; but even before the period at which his mind gave way, it is too probable that he inwardly laboured under a mental infirmity which, in itself, might be nearly allied to his genius.

The last document which we possess of Swift, as a rational and reflecting being, is given by Sir W. Scott, in his Life of the Dean; and, as Sir Walter says, awfully foretels the catastrophe which shortly after took place.

"I have been very miserable all night, and to-day extremely deaf, and full of pain. I am so stupid and confounded, that I cannot express the mortification I am under, both in body and in mind. All I say is, that I am not in torture; but I daily and hourly expect it. Pray let me know how your health is, and your family. I hardly understand one word I write. I am sure my days will be very few—few and miserable they must be.

"I am for those few days, yours entirely,

"J. SWIFT."

"If I do not blunder, it is Saturday."

From these glimpses of the misfortunes and imperfections of great men we may see that genius is not unalloyed. Carlyle, in his estimate of Scott, has much sense in the following remark:—"The truth is, our best definition of Scott were perhaps even this, that he was, if no great man, then something much pleasanter to be, a robust, thoroughly healthy, and withal very prosperous and victorious man." What we call genius has often a degree of eccentricity connected with it, and, as Dr. Ferriar has hinted, may even have an alliance with insanity. "No doubt," says he, "the same causes which in a strong degree produce madness, may, in a lower, increase the natural powers of the mind. High cultivation and extraordinary talents are not in themselves the certain means of goodness nor of happiness. The mind, rendered more subtle and more oppressed with thought and ambition, looks back even to its primitive simplicity with a feeling little short of regret. Byron says—

"I feel almost at times as I have felt
In happy childhood; trees, and flowers, and brooks,
Which do remember me of where I dwelt
Ere my young mind was sacrificed to books,
Come as of yore upon me, and can melt
My heart with recognition of their looks."*

On the other hand, the mental powers which are the farthest removed from insanity, are not perhaps the most ingenious, fertile, or ima-

* Poetical epistle written to his sister.

ginative. The more brilliant mind which sees relations not perceptible to others—the associations of which are not too strong and constant to forbid erratic flights, may be, on this very account, the more capricious, unsettled, and uncertain in its operations—possibly, the more easily pushed from its own balance, and lost from its very want of anchorage to slow and settled, albeit erroneous, doctrines. They who have undergone great changes of opinion can alone know what it is to abandon the landmarks of thought, and go out into a sea of meditation, without any well-known beacon to guide them, or familiar appearance to mark their course. Errors may be dear, *even sacred* to the mind, and to abandon the errors which ages have nursed, may indeed be difficult to those who have cherished them from their earliest infancy.

Moral Insanity.—As minds differ greatly in respect to the intellectual powers, so they differ also in regard to the affections and instinctive impulses, and these latter appear to be subject to aberrations independently of the former. The late Dr. Prichard, in a very able work on "Insanity in its Relations to Jurisprudence," has treated of the subject under the head of "Moral Insanity," and were I to state all that I think important, I should not content myself without transcribing the greater part of what he has written. It may seem rather startling to look upon *moral* aberrations as resulting from mental infirmities. I am far from wishing to appear as an apologist for vice, but a close attention to the subject obliges me in fairness to admit, that the propensities and impulses of the mind may want a just balance as well as the intellectual powers. I feel that I have not here room to do justice to the subject, but I may say that those who have given most attention to the various phases of insanity are coming to this opinion. Such views cannot perhaps alter materially our social relations nor penal institutions, but they will at least make our charity more broad and lasting, and our compassion for the erring more enlightened. Undoubtedly the protection of society requires that the morally insane be arrested in the career of crime; for if it "*needs be that offences come,* it must also needs be that there *be woe to him through whom they come.*"

The facts upon which the evidence of moral insanity rests, are too striking and numerous to admit of doubt, and the subject would probably have long ago been more fully investigated, had it not been for the privacy attached to instances of this kind, and the reluctance which is naturally felt to admit a plea which appears, at least on first consideration, to have dangerous consequences.

The existence of moral insanity is recognised by Esquirol and Georget, on the continent, as well as by Prichard, Dr. Hitch, and others, in England, indeed, by the best authorities we have on the subject. Insanity is far from showing itself merely in hallucinations and illusions, as is commonly supposed. Often the first overt act of insanity is one of moral delinquency or extravagance, and however delicate the question may appear in a moral consideration, it is impossible to shut our eyes to the truth. In many of these instances it seems that the absence of moral control constitutes the essence of the disorder; and persons are known to conduct themselves well in asylums who, immediately on being released, give way to excesses of maniacal excitement, or betray the most ridiculous eccentricities and absurdities. I may probably, on some future occasion, return to this subject; here, I can only hint at it.

INSTINCTS AND PROPENSITIES. — Independently of this moral insanity, there are also grades of difference in the passions and propensities of the mind, less excessive, but still worthy of study in connexion with such considerations as these.

That attachments are to some extent instinctive would appear from the maternal fondness even of the brute creation; and it is beautiful to witness the tender offices to which it leads. There can be no doubt but that feelings of personal attachment are much stronger in some people than in others, and may even exist to a morbid degree. Sheridan, in his play called the *Rivals*, apostrophises LOVE as the tormentor and fiend, "whose influence acting on men of dull souls makes idiots of them, but meeting subtler spirits betrays their course, and urges sensibility to madness." Dramatists and novelists have in all ages amused mankind with the tragical creations of fiction, but such stories interest only because the annals of real life have also their broken hearts.

Perhaps one of the most beautiful delineations of human attachment in comparatively modern literature, is that of Goëthe's Margaret, in his celebrated Faust. The clinging of affection in the last hour of horror and of crime rises to a sublimity which is only heightened by the confiding simplicity of the character. The mystical and unholy career of Faust contrasts with the beautiful love of Margaret, nor can there be imagined in the downward career of destruction any reproach which can speak so eloquently as the voice of unaltered love :—

"Where I have him not,

Is the grave to me,

The whole world

Is embittered to me.

For him alone look I

Out at the window.

For him alone go I

Out of the house.

My peace is gone,
My heart is heavy,
I shall find it never,
And never more."*

Attachment to place may exist with more or less strength. Some persons have strong preferences for particular places, and feel, when removed, as if transplanted to an uncongenial soil, and pine for the scenes they have left.

Amongst the Swiss, love of country sometimes rises to a degree which actually impairs the health. I think Sir Walter Scott in his last illness felt in the spirit of his own poetry a strange yearning for the scenes of his native country after literally "wandering on a foreign strand."

Hatred may be mentioned, as well as attachment, as a feeling

* From a Prose translation of Faust.

liable to morbid excess. Very strong and enduring hatreds are sometimes indicative of insanity itself, especially where they are cherished without a sufficient cause. Happiness is compatible alone with a healthy balance of the mind, and the indulgence of proud hatred and uncharitable hostility is only a small remove from the ravings of the madman, and the sound and fury which signify nothing.

In connexion with the subject of hatred, I may mention a law of the human mind which has always appeared to me of the greatest importance—I mean, that the passions are greatly augmented by the manifestation or expression of them. I have sometimes overheard an animated conversation, passing by transitions of bitterness into the most violent quarrel, each party rather fanning his own feelings by the expression of them than listening to the abuse of his opponent.

A woman will sometimes set her arms a-kimbo in the street and commence in a moderate tone of reproof, but after a time she will acquire greater and greater warmth until she flies at the cap of her antagonist. This law of the mind is very apparent in Lunatic Asylums. I remember an old woman at the County Hospital at Lancaster, who is always abusive, but the first few words she says, especially to a stranger, are tranquil enough—after a little time, however, she waxes louder and louder, and more and more vehement, although no remark or opposition may have been offered to what she has said.

A tendency to excess sometimes seems to take its origin in mental peculiarity. Where the imagination strongly predominates there is often a liability to intemperance or excess, which brings talent to contempt, and is a matter of surprise to those of more obtuse but evenly regulated minds. The fate of the unfortunate Savage has been rendered familiar to all the lovers of literature by the able pen of Johnson. Addison and Parnell are said to have drunk to excess, and Burns is well known to have given way to the same vice.

Anthony Wood says, speaking of John Milton, who, according to Erasmus, was "the light and glory of English literature," "that he was guilty of many crimes, *as most poets are.*"*

Macnish says, "Men of genius are unfortunately addicted to drinking. Nature, as she has gifted them with greater powers than their fellows, seems also to have mingled into their cup of life more bitterness. There is a melancholy which is apt to come like a cloud over the imaginations of such characters. Their minds possess a susceptibility of structure which unfits them for the gross atmosphere of human nature; wherefore high talent has ever been distinguished for sadness and gloom."

"His fame," says Carlyle, (in speaking of the literary man) "rarely exerts a favourable influence on his dignity of character, and never on his peace of mind; its glitter is external for the eyes of others; within, it is but the aliment of unrest, the oil cast upon the ever gnawing fire of ambition, quickening into fresh vehemence

* Quoted from the "Memorials of London," p. 328.

the blaze which it stills for a moment. . . . . . . The calamities of these people are a fertile topic, and too often their faults and vices have kept pace with their calamities. Nor is it difficult to see how this has happened. Talent of any sort is generally accompanied with a peculiar fineness of sensibility. Of genius, this is the most essential constituent, and life in any shape has sorrows enough for hearts so formed."*

In conclusion, I may observe, that it is impossible, for many obvious reasons, to enter fully, in this place, into the subject of medical psychology, but it does seem to me that it is one which has not yet found its due place in human consideration. It is a mistake, and a great mistake, to suppose that it can lead only to metaphysical subtleties and abstruse speculations. There are facts before us, respecting the habitudes of the human mind, which are as much to be relied on as any in physics, and as capable of being generalised and rendered available for practical ends. Ignorance of these facts is daily and hourly leading to cruel judgments, and to misery in all its various forms. I am glad to say that we have now a "Journal of Psychological Medicine" (one volume of which has already been published), and which will, no doubt, greatly aid in bringing about just views, and removing old and injurious notions. For my part, I most solemnly believe, that there are varieties of mental constitution as great as any in outward form; and when I further consider the difference which circumstances and education produce, it appears most unfair and cruelly uncharitable to measure all by the same standard, and expect like results from different minds. Yet this is the practical effect of common notions—the doctrine which is tacitly implied in our literature, and in our law. There is an old adage that what *man has done, man may do,* but it would be ridiculous logic to interpret such a passage to signify that what *one man* has done, *every man* may do.

The abstract mind—the *general term* of logicians—I grant, has no disorders, no grades of health, no varieties of power—but of this abstraction there is nothing in nature. The mind is connected with the body, and affected by the body, and there is still a lesson of charity to be learnt from a knowledge of such an association.

There is a common cry against the doctrine of different grades of mental and moral capacity, as though it impugned the goodness of the Deity; but to me it seems only in accordance with the great plan of Divine government, which commits different talents to the care of his creatures. Some men are deformed, others good-looking —some invalids from infancy, others robust—some idiots, epileptics, insane—some rich from birth, others poor—some the descendants of a long and, may be, an honourable line of ancestors, others the children of beggars and of pickpockets. The brains of men differ in shape and in volume, and shall the mind be the same in all?

It seems to me on such a subject that *not* to think *boldly,* is to

* P. 54, Life of Schiller.

think *unjustly,* and to fear the truth on such a question, is to imagine that the works of the *eternal* Creator have need to be veiled by the flimsy apologies of his perishing creatures.

Higher Broughton, Manchester.

---

### SUPPOSED DEMONIACAL POSSESSION.

